# Experiences of Serving and Ex-Serving Members With the PTSD Coach Australia App: Mixed Methods Study

**DOI:** 10.2196/18447

**Published:** 2020-10-08

**Authors:** Jane Shakespeare-Finch, Karolina K Alichniewicz, Esben Strodl, Kelly Brown, Catherine Quinn, Leanne Hides, Angela White, Gabrial Gossage, Loretta Poerio, Dimitri Batras, Samantha Jackson, Jess Styles, David Kavanagh

**Affiliations:** 1 Queensland University of Technology Brisbane Australia; 2 Ramsey Health Brisbane Australia; 3 Gallipoli Medical Research Foundation Greenslopes Private Hospital Brisbane Australia; 4 University of Queensland Brisbane Australia; 5 Royal Brisbane and Womens Hospital Brisbane Australia; 6 Department of Veterans Affairs Canberra Australia; 7 Mental Health Strategy & Research, Joint Health Command Canberra Australia

**Keywords:** PTSD, stress disorders, posttraumatic, self-management, evaluation, qualitative, health, veterans, armed forces personnel

## Abstract

**Background:**

PTSD Coach Australia is an app for serving and ex-serving defense members and was adapted for the Australian context in 2013 from PTSD Coach, which was created in the United States.

**Objective:**

This study aimed to provide a user-centered evaluation of the app from the perspective of serving and ex-serving members of the Australian Defence Force.

**Methods:**

Qualitative data were collected in response to questions to participants in 1 of 5 workshops (n=29) or in telephone interviews (n=24). Quantitative data were collected using the user version of Mobile Apps Rating Scale (uMARS).

**Results:**

Analysis of the qualitative data demonstrated mixed support for the app. While some people found it extremely useful, especially as an adjunct to therapy, others pointed out limitations and cautioned against the app potentially triggering symptoms in people with PTSD. This perceived risk was usually found to stem from frustration with the app’s functionality rather than its content. Participants spoke about the helpful and unhelpful aspects of the app and barriers to its use and made suggestions for improvement. Many participants encouraged its continued use and highlighted the need for it to be promoted more broadly, as many were not aware of it until they were invited to participate in this research.

**Conclusions:**

PTSD Coach Australia was seen in a positive light by some participants, but others thought it had too much text and the potential to trigger a traumatic response in users with PTSD. A need to update the app was also a common comment as was the need to increase awareness of the app’s existence.

## Introduction

Current research suggests that serving defense members have higher levels of posttraumatic stress disorder (PTSD) than members of the general community [1]. However, perceived stigma and concerns about potential consequences of a diagnosis of mental disorder remain particularly important barriers to help seeking among serving defense members and veterans [2,3], and self-management of symptoms is a more attractive alternative [2]. A partial solution to these challenges involves digital mental health resources. Web-based interventions for mental health issues have demonstrated benefits for self-management of a wide variety of problems, including PTSD [4]. Smartphone apps offer additional advantages over web programs because of their potentially constant accessibility and high community uptake [5,6]. While there is a wide range of mental health apps to support serving and ex-serving defense members [7], there is limited evidence about their acceptability and impact.

The United States Department of Veterans Affairs treatment providers developed the “PTSD Coach” app in 2011 in collaboration with veterans who had PTSD [8]. Subsequently, the Department of Veterans Affairs in Australia received permission to modify the app for use with an Australian audience. For example, audio was now read by people with Australian accents, Australian prevalence rates of PTSD were used, and some images were changed to reflect the Australian landscape. The resultant “PTSD Coach Australia” was jointly released by the Australian Departments of Defence and Veterans Affairs in February 2013.

There have been some evaluations of the original PTSD Coach app [9-11], including research suggesting its acceptability and benefit for self-management of posttraumatic stress symptoms [12,13]. However, it was not clear whether the Australian version of the app was well accepted and meeting the needs of Australian defense members. Hence, the aim of this research was to assess serving and ex-serving defense members’ perceptions of PTSD Coach Australia, using a combination of qualitative and quantitative methodologies. The research provided an opportunity to determine whether the app retained a high level of acceptability and perceived utility after being in use for several years and adapted to a different national context.

## Methods

### Ethical Approval

The study was approved by The Departments of Defence and Veterans’ Affairs Human Research Ethics Committee (DDVA HREC; E017/004), Queensland University of Technology Human Research Ethics Committee (1700000173), Greenslopes Research and Ethics Committee (17/17), and Townsville Mater Human Research Ethics Committee (E017-004); command approval for participation was obtained from all Australian defense forces.

### Participants

A total of 53 serving and ex-serving defense members volunteered to participate in workshops or telephone interviews. Of these 53 participants, 29 participants (n=8 serving, n=20 ex-serving, and n=1 unknown) took part in 1 of 5 user-experience workshops. Almost all were male (n=28; one indicated gender as “Other”). Of the workshop participants, 18 were from the Army, 4 were from the Air Force, 4 were from Navy, and 3 did not disclose the Australian Defence Force arm they served in. Of the 29 workshop members, 27 provided their age. The age range was 35-79 years (median 62, SD 12.4 years). Only 3 of the workshop participants were aged under 40 years. The average service time was 14.1 (SD 10.5) years. Most (24/29, 83%) had been deployed, and the average number of deployments was 2.2 (SD 2.5). Of all workshop members, 20 (69%) had been deployed to warlike operations and 15 (52%) had been in a combat role. All had experienced at least one traumatic event in their lifetime, and all but 4 (85%) had received treatment for PTSD.

Further, 24 defense members (n=8 full-time serving, n=15 ex-serving, and n=1 reservist) participated in telephone interviews. Of these, 4 (17%) were female. Of the interview participants, 11 were from the Army, 8 were from the Navy, and 5 were from the Air Force. Their ages ranged from 31 to 80 years with an average age of 55.8 (SD 12.7) years, with only 1 interviewee under the age of 40 years. The average service time was 20.4 (SD 12.8) years. Most had been deployed (21/24, 88%), and their average number of deployments was 4.0 (SD 5.6). Of all telephone interview respondents, 14 (58%) had been deployed to participate in warlike operations and 11 (46%) had been in combat roles. All 24 reported experiencing a traumatic event in their life, and 10 (42%) had received treatment for PTSD.

### Procedure

Recruitment was undertaken through the Department of Veterans’ Affairs’ At Ease website, social media, and community support advisors; Department of Defence websites and newspapers; Defense-orientated organizations such as Mates 4 Mates, White Cloud, the Gallipoli Medical Research Foundation, Defence Families Australia, and the Defence Community Organisation; and treatment and support services including Greenslopes Private Hospital, the Veterans and Veterans Families Counselling Services (now Open Arms – Veterans & Families Counselling), PTSD Trauma Recovery Group Programs, Garrison Health Services, Part Two, Career Shift, Go2 Human Performance, and private practices. The use of focus groups and interviews is closely aligned with the protocol developed by Townsend and colleagues [14] for assessing user experience of digital health resources.

### Workshops

The 2-hour user-experience workshops involved 4-10 people and were facilitated by 2 members of the researcher team, including a registered psychologist with clinical training. Initially, participants were asked to open the app or download it if they had not used it before. Those without a smartphone were provided a smartphone or tablet with the app already loaded and given written instructions on how to download them in the future. After spending approximately 20 minutes investigating the app, they completed the user version of the Mobile Apps Rating Scale (uMARS) [15], which has 4 subscales (Engagement, Functionality, Aesthetics, Information) and 4 subjective quality items (Would you recommend the app? How many times would you use it? Would you pay for this app? Overall star rating). uMARS has been found to be a reliable and valid measure [15,16].

A focus group discussion was then facilitated by one of the research team members. Focal issues for all groups included their overall experience of using the app, specific aspects of the app that were most and least helpful, what role (if any) the app may have in supporting people with trauma-related symptoms, potential barriers and enablers to its use, how these might be addressed, and how the app might be improved.

### Interviews

As conducting workshops around the country was not possible, telephone interviews were offered to increase participation from people in different geographical areas. As workshop facilitators were not on hand to assist with app download and exploration, participants who had not used the app in the past were given an opportunity to download it and become familiar with its content 2 weeks prior to the interview. This approach was also taken, as participants in interviews were likely to be using their phones for the interview, potentially the same device they were downloading the app onto. The individual 20- to 40-minute structured telephone interviews were conducted by registered psychologists with clinical training. Topics were the same as for the user-experience workshops. Interviews were audiotaped and continued until saturation of themes was obtained.

### Qualitative Data Analysis

All audio of the user-experience workshops and the interviews was professionally transcribed; themes were extracted from the data and grouped under the 6 questions posed, adhering to Braun and Clarke’s [17] Thematic Analysis approach. Analysis involved initially becoming familiar with the data through reading and re-reading transcripts and taking notes in the margins. Subsequent readings involved a process of constant comparison within the transcript and then between transcripts, combining notes into lower-order themes that were eventually grouped into higher-order themes. This approach focused on patterns of meaning across the whole group of participants and within subgroups (ie, serving and ex-serving defense members). Transcript data were supported by observational data, whereby notes were taken by facilitators about how participants were seen to be using the app.

### Expert Ratings of the App

Two raters, including the lead author on the paper regarding the development of the expert version of the Mobile Apps Rating Scale (MARS) [16], provided ratings of the app.

## Results

### Quantitative Ratings

Results of the uMARS and MARS are presented in Table 1. The app was rated moderately high on the uMARS, with its information and functionality being the most highly endorsed features. An expert rated aesthetics, subjective quality, and functionality somewhat lower than the uMARS mean for defense members, but otherwise the expert ratings were similar.

**Table 1:** Ratings of PTSD Coach Australia on the uMARS and MARS.

Subscales		uMARS^a^, mean (SD)	MARS^b^ scores - Rater 1	MARS scores - Rater 2
**Objective subscales^c^**				
	Engagement	3.48 (0.63)	3.60	3.60
	Functionality	4.09 (0.58)	3.75	3.25
	Aesthetics	3.91 (0.67)	3.67	2.33
	Information	4.10 (0.57)	4.71	4.33
	Overall mean	3.87 (0.46)	3.93	3.38
**Subjective subscales^c^**				
	Subjective quality	3.54 (0.57)	2.50	3.25
	Perceived impact	3.36 (0.97)	3.67	3.33

^a^uMARS: Mobile Apps Rating Scale (User Version).

^b^MARS: Mobile Apps Rating Scale.

^c^All uMARS and MARS items were rated on a 5-point scale: 1 - very poor, 2 - poor, 3 - average, 4 - good, 5 - excellent.

### Qualitative Responses

As the same 6 questions were posed to all workshop and interview participants, results are grouped according to the 6 questions. The themes were spoken about in every workshop group and by all interviewees.

#### Knowledge and Experience of the App

Approximately half of the participants had used the app (most interviewees, but fewer workshop participants). Participants who had used the app (some of whom had used it for years) were generally introduced to it by their psychologist or psychiatrist, or by the Veterans and Veterans Families Counselling Services (now known as Open Arms-Veterans & Families Counselling). It was seen as an app that was easily accessed and used: “*Anyone can access it, the programme, and it was easy to use.”* Most interviewees also described having a positive experience with the app, finding it user-friendly, easy to navigate, and written in a clear and understandable language.

Some participants who had never used the app spoke with some self-deprecation: “*I have no idea how to* (use it), *for idiots like myself*” and “*We’re all dinosaurs.*” Most of these non–app users appreciated that the app might be useful if they had some digital knowledge, for example,

If you know what you’re doing, it would be effortless” and “It’d be helpful for people who know how to use one (app).

Another participant said,

I think for someone who’s never used it before, I just need time to sit down on my own and go through it, and really try and understand how it all works, and then make some sort of opinion on it.

Some participants observed that the purpose of PTSD Coach Australia may be unclear, given that it contains a self-assessment tool as well as strategies to manage symptoms. For example,

...You need to define what you're going to use the application for. The application seems to me like it's got two purposes. One is to actually, ‘Do I have PTSD?’ and ‘Here are the tools I need to manage PTSD.’ To me, that can be dangerous in the fact that you don't want people to think that they have PTSD and then start manage, self-managing. What you prefer is that, um, you want people that have been diagnosed with PTSD and are using this as an additional tool to actually help manage their PTSD.

#### Helpful Characteristics

There were various aspects of PTSD Coach that were found to be helpful. One person explained that they liked the ability to put in reminders: “*...so it gives me space to separate my mental health care from trying to remember everything else.*” Others commented that they “*liked the quotes*” referring to some being from “*Janis Joplin and a Swedish proverb*” that were “*thought provoking.*” Participants generally appreciated the audios. For example, one participant said,

I think going down the road, good to listen to, but also it allows you the opportunity to do it (listen) discreetly by just having your ear phones in,” and another said, “I personally didn’t read it, I listened to it.

Others liked to read, so they could go back over material easily if needed.

Another aspect that seemed to be helpful was the ability to monitor stress:

I’ve found it useful to let me know where my stress levels are…actually are.

This feature had been used for a number of reasons, including to self-monitor. For example, if a person was not feeling great:

Why (am I) such a cranky s**t this week? And I’ll look at it and I’ll say: oh here I am, I’m up…I’m up here, um and calm down a little bit…so it was a little bit of a diagnostic tool for me

Others used the stress monitoring feature when making a claim for veterans’ support. For example,

I’ve also used it (Subjective Units of Distress) as a tool to help in my claim and they’ve actually photographed my stress levels through the app.

Many people commented positively about the amount of information. The learning function was seen to be most helpful for people who were not very aware of PTSD symptoms and how they may manifest:

…the learning function is good. It will certainly help people that don't have any understanding or exposure to PTSD, so like family members and colleagues and that sort of thing.

For those who had been in therapy, the information was not new, but could be a useful reminder. For example,

I think there’s plenty of information in there and you can spend as little or as much time in there just discovering what’s out and about

Once you drill down into every folder and sub-folder there’s a lot of info in there and it’s just stuff that most people already know but it’s good to have the reminder.

Participants also commented on the “*nice clinical feel*” to give the app credibility: “*it looks credible, it looks professional.*”

Explaining the self-assessment section was useful, an interviewee said,

…so I can go back and, and have a look at that, in terms of how I might be feeling that day, if I've got something coming up, … I guess it's a preparatory thing in my mind, to prepare myself for an environment that I may not feel comfortable in.

People reported using this element to track their well-being such as “*…* (the app) *helps identify how you're travelling.*”

Most participants who had used the app had accessed the Manage Symptoms and Self-Assessment sections. They found these helpful in managing their symptoms and to increase awareness of how they were feeling:

…mostly using it to manage symptoms, like just to remind yourself. …if you start thinking about all your crap, you can go on here and go, let's sort this out now instead of festering on it.

Features aimed at assisting relaxation and sleep were generally seen to be positive:

I’ve used it a fair bit just for sleep and relaxation…some of the sleep coaches and the relaxing sounds, and just grounding techniques and stuff like that.

One participant said,

I found it very straightforward, very intuitive…the font was great…the spoken instructions, and the sort of coaching was all very clear.

Another participant said,

…look honestly, a very useful tool, I've done it twice last week, and I'll probably do it again later today, and on those occasions where I think I might be feeling a bit down, I might use it, as well.

Although some older participants in the workshops struggled with the technology, most of the older participants in the interviews found the app easy to use. For example, one older participant said:

…I was quite surprised when I went through this app, at how friendly it was. So, first up, it's a very friendly app. It's very easy to use. Pretty straight forward.

This difference may in part have been due to interview participants having time to download the app pre-interview, whereas some workshop participants had never used technology before, let alone an app.

Consistent with the workshop data, some interview participants said that the app was not perceived as a standalone tool to manage symptoms of PTSD or to replace a mental health professional. Rather, it was seen as more appropriately used to reinforce learning and strategies introduced in therapy sessions.

So, there's the app itself, I don't believe (it) is a sole source of solution.

…it's a good tool to help as well. I still think, you know, depending on the severity of the PTSD, they would need counselling and they would need, well, professional help…but it's a great little pocket tool to be able to you know, look at in times of crisis when you can't necessarily see a professional immediately.

#### Least Helpful Characteristics

Some participants did not like the app or found it unhelpful and frustrating when needing a quick strategy in a crisis situation. For example,

Um, it became extremely frustrating. So then knowing how sometimes I feel when I have an attack, that wasn't good. It kept going skip, next, skip, next, skip, next. And I thought, well it's not really giving me any help.

I think when you're angry, reading something, and especially if you've got to read it, understand it, and put it into practice, is not the best way to do that. …when I am annoyed, I have to read something two or three times before my brain comprehends it.

Consistent with views expressed in the workshops, some interviewees with PTSD thought aspects of the app would trigger their symptoms:

…with PTSD, you, it's a high degree and an instant degree of triggered frustration.

Some participants experienced technical glitches with the app when they had difficulty downloading it onto their device, uploading songs and pictures, and listening to some of the audio. For example, one participant said,

Bearing in mind that if you're vaguely thinking about this thing, it's gotta be a smooth entry, which in my case it wasn't.

Some ex-serving defense members who had experience using apps and in particular, PTSD Coach Australia, voiced concerns addressing technical issues such as flexibility and the potential to personalize the app. For example, one participant’s perception was as follows:

I wanted to write a doctor’s appointment (in it), there was no way for me to put it in.

Another user agreed that the app “*doesn’t utilise all the capabilities of my* (brand of phone).” Lack of flexibility was a widespread theme:

Learn (from menu) is very important, that should be at the top, but for someone who has been using it for some months or years, they should be able to drag the learning component down at the bottom.

A history function could also be used as a reminder of what a person was doing when they had a good day: activity icons could cue memories and be selected instead of reading and typing information in about activities undertaken during a good day. Some people suggested that the app was not as personalized as they would like and that different audiences (age groups, sex) needed different information or information communicated in different ways. If it were “*...individualised and I can go – oh yeah that, that really resonates with me,*” that was expected to increase uptake. Essentially, the app was seen to have outdated functionality when compared with current phone and tablet technology.

Some issues with the stability of the app’s functions were identified. One person said,

I was just trying to set reminders for things then (during a workshop) and I don’t know if it’s something to do with my phone but it just keeps shutting down every time I try and set a reminder.

Further probing revealed the person was using a particular brand of Android phone, and some other users of the same brand of phone had the same experience. During the workshops, facilitators also noticed that histories were sometimes lost. For example, a participant did the assessment task, but the app would not let the user see the results—“*I don’t know where it went. Just disappeared.*” This was frustrating, because using the app to monitor their health was seen as important.

Some participants saw the app as an agent of authority. For example,

In the first paragraph it says… you agree to the terms and conditions. I went right through that front page, and nowhere is there a hidden panel or any sub-element of terms and conditions…I love how authority works.

Another said the tone was suited to serving members because “*they’re telling you what to do, and it’s like really, like being back in the army with your sergeant…*” and a third explained, “*as soon as you get out* (of the military) *you will not be told what to do.*” Essentially the app was seen as *“very militarized,*” and that was a connection that many ex-serving members did not want.

Many people spoke about the density of information, for example: “*I found the menus are wordy, lengthy, boring.*” Color coding was a suggestion to “*make it a little more accessible*” such as having different colors for health, learning, and self-assessment. Others suggested increasing the density of information as a user progressed through the layers of the app:

The first layer, so the initial layer, entry layer looks good, then your second layer, then once you step into the sub-elements, so tertiary and beyond, it then gets very overloaded.

It was pointed out that this level of complexity and denseness of information was not helpful for people with a mental disorder. When becoming overloaded a person would “*switch off and not really care about it.*” Some people found that the denseness of information elevated their emotional arousal:

There’s a lot of reading in there, and I switched off. You know, black and white, black and white, read, read, read, reference to X, Y, Z. I don’t care about X, Y, and Z, what does this bloody thing do? … so, I switched off and went away, and had to calm down and then come back to it. It made me angry.

For some people, the Self-assessment section seemed to evoke distress. For example,

So I used the self-assessment and I got halfway through it and the questions were actually, for me they started to become disturbing, so I stopped.

The Learn section of the app was seen as not thorough enough by some interviewees:

...I must say my first reaction to it (Learn section) was, I thought, oh, this is pretty superficial, you know, what's PTSD, and how it develops, and how common is it…and the stuff like that.

Others identified Soothing Pictures and Soothing Audio as least helpful:

Now I've been through those resources that are listed, and basically found them, for me anyway…no use.

As already noted, some participants found the inspiring quotes useful, but others did not; for example:

Ah, yeah, it's the last thing I want (laughs)…I'm not into those touchy-feely things.…I suppose it's just the word 'inspiring' sort of put me off straight away.

There were also a number of comments about the app’s design:

(The) one that comes to mind most of all is the computer-generated voice. …I found that to be, uh, um, not easy to understand, and also very impersonal.

One person found “*some of the text is actually overlaid on top of the scroll bar, the tool bar at the bottom.*” Another said: “*Once again, too much information. Text is too small.*” This view was commonly asserted in both the telephone interviews and the workshop groups and was linked to unwanted emotions. For example,

…to navigate through and looking at a small screen, I, if I spend more than 20 minutes, I just end up getting really angry with it.

#### Potential Role of the App in Supporting People With Trauma-Related Symptoms

Most participants who had used the app had done so with the help of their clinician. PTSD Coach Australia was seen by them as a positive adjunct to therapy: *“…more helpful if you are seeing a psychologist or psychiatrist.*” However, as already mentioned, many participants commented about the potential for material to retrigger their symptoms. For example,

You want to be careful you don’t stir it all up…reliving it and rehashing it and all that. You know?

Some people did not think using the app would “*push people to get help,*” whereas others thought recognizing symptoms through the app may spur people on to seeking help: “you say, s**t this is happening to me so you’re going to do something about it.” Many of the older participants thought it unlikely that they would access an app or that others from their cohort would, stating that when a person is experiencing symptoms of anxiety or depression “*you’ll want to talk to somebody that’s going to give you advice…*”

The dangers of self-diagnosis were also raised:

People self-diagnose and they read the symptoms and say, yeah I have PTSD. You really have to go see an expert and psych to get information. You shouldn’t do it yourself.

A perceived lack of consistency in the content of the app with what clients were taught about PTSD and its management in therapy was also discussed as a retriggering feature:

Well you know, me personally, I’ve come a long way from hating the world, and I’m comfortable with where I am now, and then I come in here and this (the app) contradicts what I’m being taught, which has made the world a better place for me. So that makes me confused, angry, emotional, frustrated.

#### Potential Barriers and Enablers to the Greater Use of the App

The main enabler to the greater use of the PTSD Coach Australia was that it is available for free, which was good “*because I wouldn’t pay for it.*” A barrier for some was their lack of familiarity with technology in general and apps in particular. Some suggestions were offered to make access easier:

Get the RSL [Returned Services League] to set up trainee workshops through their sub-branches…work in a group and kind of demystify it because it’s actually quite simple when you know the trail.

For many older (ie, Vietnam veteran) participants, the technology was foreign and using it was overwhelming.

Others pointed out that an app of this sort would be difficult to negotiate if they had additional physical complications such “*as a stroke or a chronic illness such as Parkinson’s.*” Another suggested the app be available in “*A DVD that you can just stick in…and watch and listen to it.*” These quotes came from older participants and may further indicate discomfort with the technology. A lack of visible marketing of the app was noted.

The name of the app was raised by many participants, and the icon was also discussed. There were 2 issues in this theme. The first was that the app’s title indicated that it was for people who have PTSD, but that the app could be used by a much broader population. The second issue concerned stigma. Having an icon on their device that others may see (eg, on the train or bus) that suggests they had PTSD was considered to reveal personal information. Stigma regarding mental health was still seen as a problem, and more education was needed:

If we can educate our new members and our current members, well, you know, there is still a lot of prejudice against mental health (problems) within Defence.

The name of the app did not seem to be useful in breaking down that stigma. When asked what would stop someone using the app, one participant summed up the sentiment as, “*shame, sense of shame.*” When asked about potential barriers to use, others said,

Just its name, PTSD Coach. I don't know what you could call it, but people don't want to be labelled.

My nephew wouldn't download it. My friends wouldn't download it. Uh, most people won't download it if it's called PTSD coach. …if you call it Wellness Coach…

Other suggestions for renaming it included “*Mental Health Coach*” but some doubted whether that would reduce perceived stigma. One suggested that the information screen “*could say this can help if you are having trouble sleeping, if you’ve got anger issues, blah blah…*”

Many participants stated that they could not find any other barriers or challenges in using the app, as it was informative and well set up: “*…I haven't found it to be difficult at all. It's just been interesting…it's easy to read, it's easy to access and it's easy to understand.*” Another participant said, “*No barriers. I was fine. I can see that the app is useful …when it's* (used) *in conjunction, obviously, with some sort of help as well.*”

#### Ways to Improve the App

The majority of participants stated that no improvements to the app were needed: “*I think it's great. I think, for what it's meant to do, I think it's fantastic.*” However, others suggested that including more strategies and interventions such as crisis intervention, mindfulness, mood tracking, and elements of exposure therapy would be beneficial: “*So, as it stands now, the app can assist, but it wouldn't be able to assist through a crisis moment.*” Another common subtheme was that participants suggested expanding information in the Learn section, for example, by including links to additional information, including videos with psychoeducation and presentations of techniques:

(Include) a hyperlink to a webpage or something that may be able to help people if they want to do further research.

There should be, uh, there should be video examples, rather than trying to find written descriptions of how you deep breath.

Most participants noted the need for increased awareness about the app and the need for its promotion to assist serving and ex-serving defense members who had experienced trauma:

…how it can be used is by making people aware that it exists, seriously. I think that's the priority

…to actually send out a thing that says, “Here's a link to an app, and you can download it, and it doesn't cost you anything…”

One person who described himself as belonging to the younger generation said,

...it probably wouldn’t hurt to have a YouTube video explaining what the purpose of it (is) and how to use it and how to maximise the benefit out of it.

Rather than a video explaining how to use the app, others suggested

…just a little video clip, a YouTube like video clip on it (the app itself). Nothing that takes up space or time, but just enough to get the gist of what’s going on…maybe the odd skit.

Using the app as a way of connecting with peers was suggested. When speaking about the possibility of the app being a platform for that purpose, one participant explained,

A group amongst veterans is the strongest form of medicine you can have, the one-to-one with your peers.

This potential function may also be a way of keeping track of each other; if a person had not heard from a peer for some time, it may prompt them to contact them to check how they were going.

Although many people liked the fact that they could load their own music and pictures, others suggested that “*maybe having a library in there of pictures and sounds rather than having to upload your own into the system.*” A younger participant said that he “*liked that you could alter and change images to what you like. I had a bit of fun like, putting all my kids in.*” However, there were some restrictions in the functionality of the app when trying to alter images for things like relaxation, for example, the app “*doesn’t allow you enough freedom to perhaps get something else off the internet without you having to do a separate action and then upload it to the app.*” Others found some of the images insufficient; they were perceived as “*a little bit sterile.*” One participant was specific in his feedback about some of the images:

…a picture of a person’s head with brain cogs, okay it was clear, but I find it a bit offensive actually. It’s like…I’m on the app that’s gonna help me deal, I don’t want to feel like I’m being analysed or I’m a nut case, or a statistic by the other icon with a line coming up and a lightning bolt going down.

Some people remarked that the images on the app were not relaxing to them:

There is a dog in a purple field in a yoga position…what I find is that is not relaxing, it’s sort of more something like, you know, something a hippie would use.

## Discussion

Many responses to PTSD Coach Australia by the serving and ex-serving defense members who participated in this research were positive, with uMARS ratings in the “average” to “good” range and many positive comments. Most participants said the app was user-friendly, easy to navigate, and helpful in managing symptoms of PTSD. This feedback was consistent with studies evaluating the American version of the app [8,13,18,19]. Key components of the app (eg, Manage Symptoms, Find Support, and Learn) appeared to be maintaining the acceptability and perceived benefit that were observed in earlier research on its American version [18], and many participants continued to value the ability to track changes in stress or other symptoms. However, scheduling of reminders appeared less valued by some users than in past studies [12,18]. Inspiring quotes were generally but not universally valued.

Some participants suggested its use as an adjunct to face-to-face treatment rather than as a stand-alone tool. Consistent with broader evidence on the benefits of having a therapist or coach to guide and support the use of digital mental health tools [18], a pilot study comparing self-managed versus clinician-guided use of PTSD Coach found that clinician support appeared to increase the app’s symptomatic benefits [4], although both modes of application improved PTSD symptoms. Some participants also expressed concerns that self-managed use of the app may generate frustration or trigger trauma symptoms in some users and may not be well suited to crisis management. Improvements to the app’s functionality and reduction of text could avoid triggering frustration and distress in vulnerable users. A recent systematic review and rating of apps designed to assist with PTSD [20] noted the absence of safety planning in the PTSD Coach and PTSD Coach Australia apps. While the app has a listing of crisis services and an ability to set up individualized support contacts, additional safety planning within the app may be indicated.

Consistent with previous US research [8], many participants reported that the app was easy to use, although some wanted faster navigation to favorite tools. Many now found the app to have suboptimal functionality, have some out-of-date content, and lack engaging graphics and flexibility. These observations may reflect changes in user expectations. Importantly, current participants also experienced significant technical problems when using the app on some Android devices, suggesting that its updates had not kept up the pace with developments in devices and software. Interestingly, previous research suggested that the app would appeal even to technology novices and older individuals [8], whereas (despite the passage of time) current respondents expressed the view that people who had little experience with digital technology may struggle with its use (and some saw this as applying to themselves). Some also noted that use of the app would be difficult for people with a physical disability or cognitive deficit and for those experiencing a heightened state of emotional arousal. Applying recent advances in app design and functionality could help address these issues.

While the availability of PTSD Coach Australia free of charge was seen as an enabler of its use, participants noted a lack of awareness of its existence, suggesting that greater promotion of the app was needed. Some participants proposed that this and other mental health apps could be discussed and used prior to deployments to assist with stress management.

A limitation in the current research was that the participants were predominantly older than the average age of currently serving personnel, which may limit the representativeness of findings in relation to use of the app by younger people. There was also a wide range of digital literacy regardless of age. A difference in the procedures used for interviews and workshops could be seen as a limitation: Interviewees were asked to download the app prior to the interview due to the likelihood of using the same device for the download as they used for the interview, whereas workshop participants examined the app at the start of the workshop, giving facilitators an opportunity to observe any difficulties they had in downloading or using the app. This difference in procedure may have resulted in workshop participants having a more recent experience with the app, although themes were consistent across the two approaches to data collection.

Overall, participants attested to the value of PTSD Coach Australia, and in particular, its potential benefit to both serving and ex-serving defense members who were receiving treatment for posttrauma reactions, and for those with less severe problems. While the app could be improved further, its current functionality was viewed mostly positively. A change in its name and increased promotion of the app could substantially improve its uptake and impact, in addition to an update of aesthetics and design to bring it in line with modern apps.

## Abbreviations

MARS: Mobile Apps Rating Scale
PTSD: Posttraumatic Stress Disorder
uMARS: Mobile Apps Rating Scale (User Version)
